# Epigenetic regulation of asthma and allergic disease

**DOI:** 10.1186/1710-1492-10-27

**Published:** 2014-05-28

**Authors:** Philippe Bégin, Kari C Nadeau

**Affiliations:** 1 Allergy, Immunology, and Rheumatology Division, Stanford University, 269 Campus Drive, Stanford, California, USA

**Keywords:** Epigenetic, Asthma, Allergy, Atopy, Inheritance, Transgenerational, Methylation, Histone, Th2, Amplification hypothesis

## Abstract

Epigenetics of asthma and allergic disease is a field that has expanded greatly in the last decade. Previously thought only in terms of cell differentiation, it is now evident the epigenetics regulate many processes. With T cell activation, commitment toward an allergic phenotype is tightly regulated by DNA methylation and histone modifications at the Th2 locus control region. When normal epigenetic control is disturbed, either experimentally or by environmental exposures, Th1/Th2 balance can be affected. Epigenetic marks are not only transferred to daughter cells with cell replication but they can also be inherited through generations. In animal models, with constant environmental pressure, epigenetically determined phenotypes are amplified through generations and can last up to 2 generations after the environment is back to normal. In this review on the epigenetic regulation of asthma and allergic diseases we review basic epigenetic mechanisms and discuss the epigenetic control of Th2 cells. We then cover the transgenerational inheritance model of epigenetic traits and discuss how this could relate the amplification of asthma and allergic disease prevalence and severity through the last decades. Finally, we discuss recent epigenetic association studies for allergic phenotypes and related environmental risk factors as well as potential underlying mechanisms for these associations.

## Introduction

The term epigenetics was coined by C.H. Waddington in the 1950’s to describe means in addition to genetics to explain cell differentiation [1]. The concept of epigenetics was initially limited to cell differentiation from pluripotent stem cells to unipotent well differentiated cells, but the modern definition of epigenetics has been broaden beyond differentiation to include non-sequence inheritance. Epigenetic mechanisms have been shown to regulate many genes including those involved in inflammation and the immune response and to ensure inheritance of phenotype with cell division [2,3].

The purpose of this review is to provide allergy/immunology professionals and researchers with a broad, yet easy-to-follow, review of the epigenetic regulation of asthma and allergic disease. The main focus will be on DNA methylation and histone modifications, their relevance in the process of allergic sensitization, their impact on disease heritability and association with environmental exposure and allergy phenotype. MicroRNA, which constitute a distinct epigenetic mechanism, are beyond the scope of this review. Their role in allergic disease has been reviewed recently elsewhere [4].

## The basics

**Figure 1:**
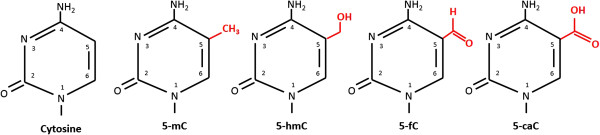
**Structure of methylcytosine and its by-products.** 5-mC = 5’methylcytosine; 5-hmC = 5’-hydroxymethylcytosine; 5-fC = 5’-formylcytosine; 5-caC = 5’-carboxymethylcytosine.

DNA methylation was the first epigenetic mechanism recognised and the one that is most extensively studied. De novo methylation occurs in response to various cellular stressors and signal by DNA methyltransferases (Dnmt3a and Dmnt3b) which add a methyl group to position 5 of cytosine residues at a CpG site (Figure 1). CpG sites are dinucleotides consisting of a cytosine and guanine (the “p” stands for the phosphodiester bond linking the 2 nucleotides) which occur throughout the genome but may be concentrated in clusters referred to as CpG islands found at important regulatory sites, such as promoter and enhancer regions [5]. CpG islands are defined as a region with 200 base pairs containing an observed-to-expected CpG ratio that is greater than 60 [6,7]. In terminally differentiated cells up to 90% of genome CpG sites are methylated with most unmethylated CpG islands found in functionally active genes [5].

**Figure 2:**
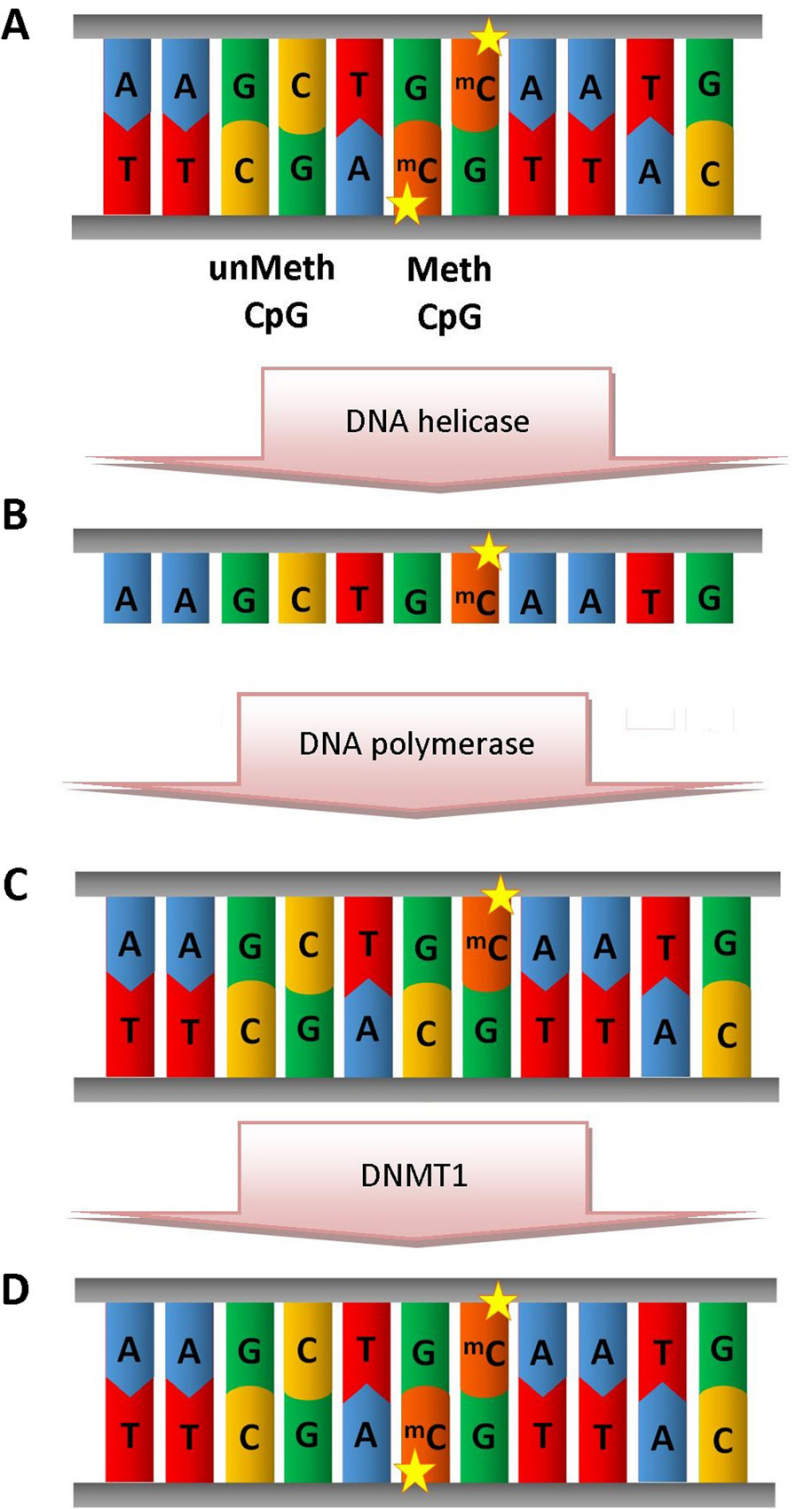
**Replication of methylated DNA.** The palindromic nature of CpG sites is key to their inheritance **(A)**. With replication, each separated strand carries one methylated cytosine **(B)**. The daughter hemi-methylated DNA **(C)** is recognised by DNMT isoform 1 which methylates CpG sites on the new strand using the old one as a template **(D)**.

The palindromic nature of a CpG site is important as it ensures replication of the methylation pattern with each cell division [5]. With DNA replication, both separated strands of DNA will each carry one methylated cytosine to be used as a template for duplication (Figure 2). The resulting daughter DNA duplex strands will thus be hemi-methylated. This hemi-methylated DNA is recognized by a different DNA methyltransferase isoform (Dnmt1) which methylates CpG sites on the new strand using the old one as a template. This maintenance methylation ensures conservation of the methylation pattern during cell division.

Conversely, a CpG site can be demethylated by oxidation of the methyl group. Physiologically, this process is initiated by the enzyme Ten-eleven translocation (TET) dioxygenase which gives rise to 5-hydroxymethylcytosine and then to 5-formylcytosine and 5-carboxylcytosine [8]. These modified nucleic acids can also be generated, although far less efficiently, by radical reactions involving hydroxyl radical and one-electron oxidants. They are then excised by the DNA repair enzyme thymine DNA glycosylase and replaced by a normal cytosine [9].

The mechanism by which DNA methylation is associated with gene silencing is still not fully understood. Earlier studies reported that methylation could directly limit the access to transcription factors (TF) [10]. Although this is true for some TF, it is not an absolute rule as some TF have been shown to have greater specificity for methylated binding motifs [11]. Methylated DNA can also recruit methyl-CpG binding proteins which compete with TF for access to binding sites [12]. Some of these proteins, such as MeCP2, can further recruit histone modifying enzymes to add another level of epigenetic modifications (discussed below) [13]. However, the interaction between DNA methylation and other epigenetic mechanisms is not unidirectional as histone modifications can also affect DNA methylation [14]. In fact, studies in stem cells and thymocytes have shown chromatin inactivation by histone and chromatin modifying enzymes to precede de novo DNA methylation during progressive epigenetic silencing [15-17]. Regardless of its underlying mechanism, DNA methylation is important in itself and should not be viewed as an epiphenomenon of other epigenetic mechanism as DNMT mutants display a multitude of defects, including aberrant gene expression, activation of mobile DNA elements and reduced genome stability [18].

**Figure 3:**
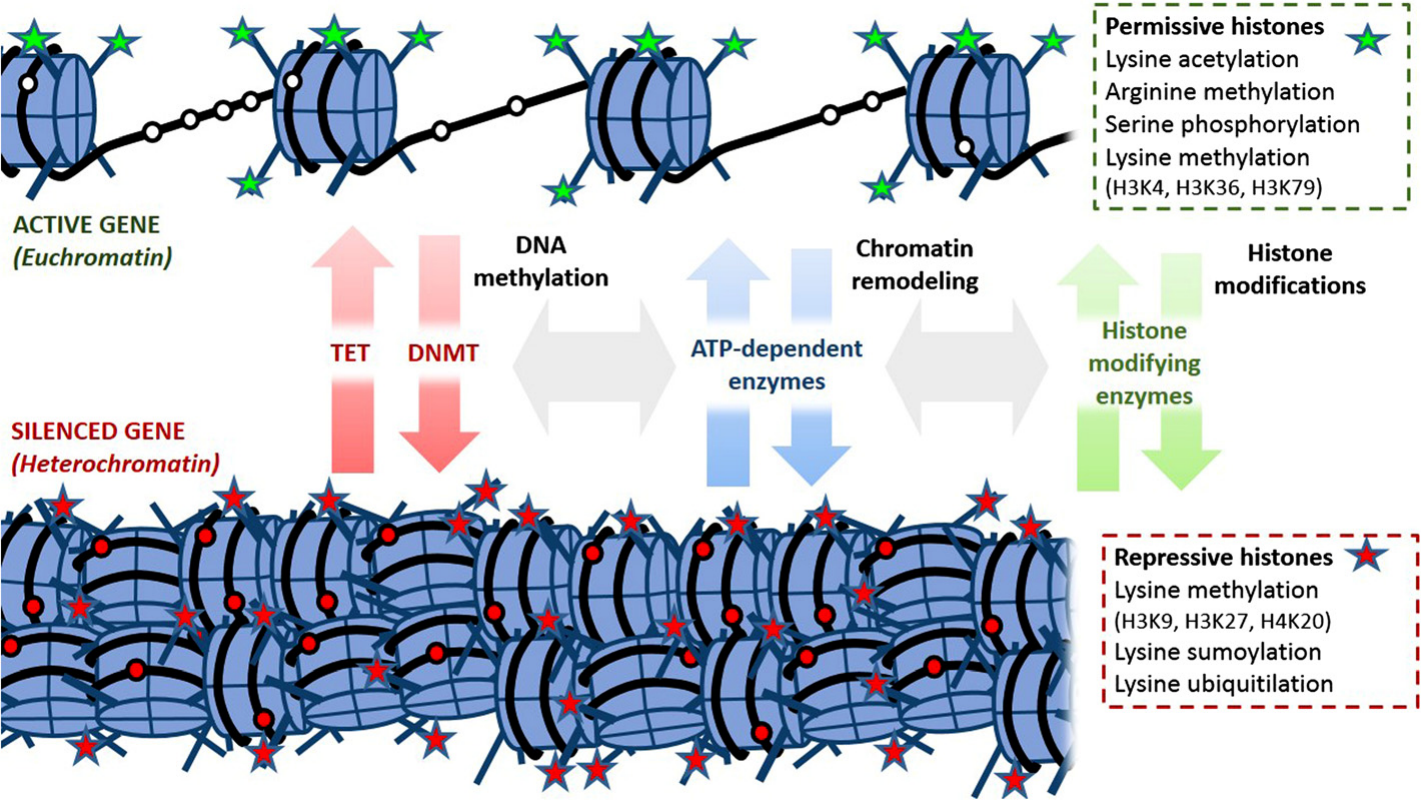
**Euchromatin and heterochromatin.** Unmethylated CpG islands (blank circles), permissive histone modifications (green stars) and loose chromatin structure promote gene transcription in the euchromatin state. Conversely, DNA methylation (red circles), repressive histone modifications (red stars) and condensed structure prevent transcription in the heterochromatin state. Although governed by distinct enzymes, cooperativity and interaction between the different epigenetic modifications provide a self-reinforcing mechanism for epigenetic regulation. TET=Ten-eleven translocation dioxygenase; DNMT=DNA methyltransferase.

Chromatin, the complex of DNA and nucleic proteins in the nucleus, is another central target of epigenetic modifications. Transcriptionally inactive heterochromatin is packed densely whereas active euchromatin is less condensed (Figure 3). The core component of chromatin is the histone octamer which organises DNA in structural units called nucleosomes [18]. The histone octamer consists of 2 dimers of core histones H2A and H2B and 2 dimers of core histones H3 and H4. Chromatin remodelling is a fundamental mechanism for establishing somatic cell memory of gene expression pattern. It is a dynamic process which is regulated by histones and ATP-dependent chromatin remodeling complexes which either move, eject or restructure nucleosomes. The "open" or "closed" state of the chromatin near a particular gene can be revealed by examining DNA sensitivity to the enzyme DNAse I by way of a procedure known as a DNAse sensitivity assay. This technique is based on the fact that DNAse I degrades open DNA more quickly than closed DNA, hence the term DNAse I hypersensitivity site (DHS) [19].

Core histones have long N-terminal tails protruding from the nucleosome which can undergo posttranslational modifications that alter their interaction with DNA and nuclear proteins. The standard way of reporting those modifications is by naming the histone, followed by the amino acid, and the modification. For example, H3K4me1 would denote single methylation (me1) of lysine 4 (k4) on histone 3 (H3). Research has shown a strong relation between covalent histone modifications and gene expression [18]. As a general rule, histone acetylation or phosphorylation are associated with an active state. Histone methylation, on the other hand, appears to have diverse function in the control of gene activity, depending on the amino acid and the number of methyl- groups added. The nature and combination of these changes determine the extent of expression, with those highly expressed genes associated with greater permissive histone modifications and those less frequently transcribed ones associated with repressive changes and more tightly packaged chromatin, although the relationship between gene expression status and histone modification is not absolute [20]. In addition to influencing chromatin structure, recruitment of chromatin-remodelling complexes by covalently modified amino acid on histone tails may also help target gene locus for pre-initiation of transcription gene [21,22]. The addition or removal of the various chemical elements on histones is catalyzed by histone modifying complexes such as histone acetyl transferase (HAT) and histone deactetylase (HDAC) which add and remove acetyl- groups on histone residues, respectively.

## Epigenetic control of T cell phenotype

### Th2 differentiation

While epigenetic changes have been coined as the hallmark of cell differentiation, their importance in other processes is now coming to light. Of note, T cell activation and skewing, which could be viewed as a certain type of cell differentiation, is governed in great parts by epigenetic changes which insure that the clone of a T cell will retain its phenotype (Th2, Th1 or otherwise) [23].

Th2 skewing is triggered by simultaneous TCR and IL4 receptor activation, which leads to the phosphorylation of STAT6 and expression of Th2 master regulator GATA-3 and Th2 signature cytokines, including IL-4. Th1 differentiation is similarly triggered by simultaneous TCR and IL-12 receptor activation, phosphorylation of STAT4 and expression of Th1 master regulator TBET and Th1 signature cytokine INF-γ, with silencing of Th2 cytokines.

In resting CD4 T cells, both IL-4 and IFN-g genes are methylated [24]. Upon allergenic sensitization, the IL-4 promoter in allergen-specific T cells is demethylated, the extent of which correlates with IL-4 expression [24]. The IL-4 locus of Th2 cells is also marked with permissive histone modifications H3K4me which are absent in Th1 or naïve T cells [25]. Similar modifications are found at the IFN-γ locus in Th1 cells or the IL-17 locus in Th17 cells.

**Figure 4:**
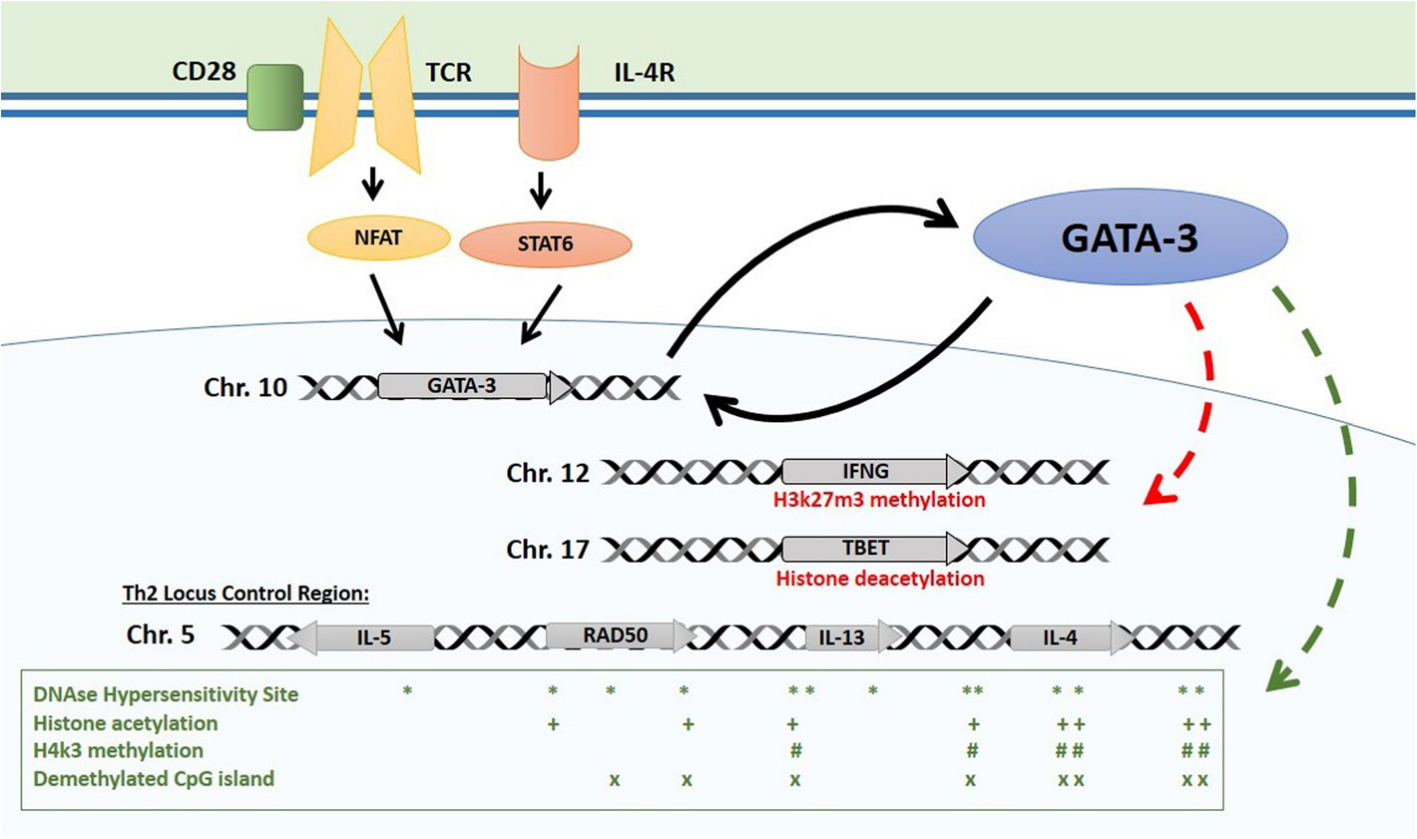
**Epigenetic control of the Th2 locus.** Master regulator GATA-3 is induced by TCR and IL-4 receptor activation and maintains its own expression with a positive feedback mechanism. GATA-3 induces repressive histone modifications at Th1 loci (TBET, IFNG). It interacts with HAT enzyme p300 with chromatin remodeling complex component Chd to induce permissive histone and chromatin changes at the Th2 LCR. Distribution of main epigenetic marks at the Th2 LCR [23] are presented in the lower box.

The main Th2 genes are positioned in the Th2 locus control region (LCR) on chromosome 5 which forms a chromatin hub that interacts with GATA-3 (Figure 4) [23]. GATA-3 then interacts with HAT enzyme p300 and with chromatin remodeling complex component Chd to induce permissive histone and chromatin changes at the Th2 LCR [26]. The GATA-3/Chd complex also binds HDAC to repress the tbx21 locus encoding TBET, the master regulator of Th1 differentiation which activates Th1 genes and suppresses Th2 genes [26], and recruits the H3k27m3 methyltransferase EZH2 to the IFN-g locus, causing its inhibition [27]. Further suppression of Th1 cytokines is achieved by the increase of their DNA methylation from naïve state [28]. In Th1 cells, STAT4 and TBET have been shown to exert similar but inverse influence on IFN-g and Th2 genes epigenetics to promote Th1 skewing [29].

The exact mechanism of DNA demethylation of Th2 genes is still incompletely understood, possibly due to the only recent discovery of the TET enzymes which are responsible for physiological DNA demethylation. In a mouse study, GATA-3 was insufficient to induce DNA demethylation of the RAD50 DHS site 7 in the Th2 LCR, although the process was shown to be dependent on STAT6 [30]. Interestingly, knocking-out RAD50 DHS site 6 prevented DNA methylation of IL-4, IL-5 and IL-13, suggesting an interdependence between those genes and an important role for the chromatin hub structure of the Th2 LCR (Figure 4) [31].

Interestingly, the GATA-3 promoter has been shown to keep its repressive histone modification despite TH2 activation and present a bivalent state with both repressive and activating histone modification [25]. This suggests an important role for positive feedback to additionally insure its stable expression, with GATA-3 positive binding to its regulatory elements. It is also worth noting that the main anti-Th1 effect of GATA-3 is exerted through direct inhibition of the IL-12/STAT4 and RUNX pathways [23].

### Establishment of T regulatory phenotype

T regulatory cells are a subset of T cells which suppress the inflammatory response and thus play an important role in immune tolerance to self and exogenous antigens. Their function and number in tissue has been shown to inversely correlate with allergic phenotypes and their importance in allergic disease has been well described [32].

FOXP3 is the master regulator for regulatory T cells (Treg) [33] which can be divided in two subsets based on their origin: Tregs of thymic origin (tTreg), which were previously referred to as natural Tregs and peripherally-derived Treg (pTregs). FOXP3 expression is controlled by proximal promoter and intronic regulatory elements designated as conserved non coding sequences (CNS1-3) which are highly conserved between species.

In the thymus, tTregs are induced by TCR engagement with self-peptide major histocompatibility complex with specific strength and duration. The subsequent NF-κB signaling induces permissive histone modification (H3K4me1) at the CNS3 and potentially initiates chromatin remodeling in the FOXP3 locus through the c-Rel subunit [34]. In parallel, cAMP response element-binding protein (CREB) binds to the CNS2 element, which inversely correlates with the methylation status of CpG islands [35]. DNA demethylation of the CNS2, also called the Treg-specific demethylated region (TSDR) is a major event in tTreg differentiation and carries an important function in FOXP3 stabilizing FOXP3 expression [36]. CNS2 is the site at which FOXP3 binds to its own gene to maintain expression in a positive feedback mechanism allowing for a persistent phenotype and suppressive function. FOXP3 induces the expression of IL-2 receptor CD25, which activation phosphorylates STAT5 which binds the promoter and CNS2 independently of methylation status providing an additional positive feedback mechanism [37].

Besides Tregs, activated T cells also express FOXP3 upon TCR engagement [38]. However, this expression is only transient as the CNS2 remains methylated. In fact, when comparing tTregs to FOXP3+ activated effector T cells there are hundreds of loci throughout the genome which show demethylation and correspond to binding sites for FOXP3 [39]. These methylation changes are not induced by FOXP3 but rather allow FOXP3 to access its targets and exert its function. The lack of demethylation of these loci could explain the difference in function despite the expression of FOXP3 in activated T cells.

In contrast to thymic-derived Tregs, generation of Tregs from peripheral naïve T cells is favored by suboptimal TCR stimulation in the presence of TGF-β [40]. TGF-β promotes FOXP3 transcription in peripheral CD4 T cells through binding of SMAD3 at CNS1 [41]. A FOXP3-CNS1-deficient mice mouse has shown that CNS1 is critical for FOXP3 induction in peripheral CD4 T cells but not in thymocytes. Interestingly, those mice lacked the self-reactive auto-immune manifestations observed in FOXP3 deficient *scrufy* mice, but had maternal-fetal conflict and inflammatory disease at the mucosal interface, suggesting a specific role for pTregs in acquired tolerance to exogenous antigens [42]. The generation of TGF-β-induced Tregs can be augmented by the addition of retinoic acid, which has been shown to induce histone acetylation at the CNS1 region [43]. While in vitro differentiated Treg cells appear to lack TSDR demethylation, in vivo pTregs gradually demethylate the TSDR which could contribute to phenotype stability [44].

## Inheritance of epigenetic traits

Development of allergy and asthma is determined by interplay between environmental and inherited factors, the later accounting for over half of the risk [45]. Interestingly, this is in high contrast with the low fraction of variance in asthma prevalence (4%) that can be accounted for by genetic loci in a large-scale genome wide association study [46,47]. This missing heritability could be due in part to the difficulty of accounting for rare polymorphisms with a high penetrance in some families (private mutations) but it also raises the possibility of non-genetic means of inheritance [46].

Genetics also fail to explain the sudden rise in allergies and asthma as even with significant selection pressure, any change in population genetics would necessitate multiple generations to occur. Epigenetic changes on the other hand can be induced much more rapidly with various environmental exposures and, like with genetics, these changes can be passed down from parents to offspring. There are several ways in which epigenetics can influence phenotype inheritance, including gene imprinting, in utero modifications and transgenerational inheritance.

### Parental imprinting and maternal influence

Asthma and atopy are complex genetic traits meaning that the phenotype is the result of the interaction of multiple genes each with their own Mendelian pattern of inheritance. The expected result is that no clear pattern of inheritance would be discernable, with risk from both parents being very similar overall. In reality, the risk for allergy and asthma inherited from the mother is up to 5 fold greater than the paternal risk [48-51].

The discrepancy in parental risk could be explained in part by parental imprinting. Parental imprinting is a process by which some genes are epigenetically silenced during gametogenesis in a parent-of-origin-specific manner, which results in only one allele being expressed for the imprinted loci. The best example probably consists of polymorphisms of FcϵR1-β which are only associated with atopy when the risk allele is inherited from the mother in multiple cohorts [49,52,53].

A more recent study showed that maternal but not paternal atopy predicted the expression profile of 18 cytokines and chemokine in the airway mucosal fluid of newborns [54]. However, it is unclear whether this is the result of true genomic imprinting or from a direct modification of the foetal immune system by the mother’s atopic phenotype in utero. Animal studies have shown that challenging previously sensitized mice to ovalbumin during pregnancy resulted in an increased allergic phenotype in offsprings [55]. Transferring T cells from sensitized to naïve pregnant mice had the same effect, suggesting this in utero influence was mediated at least in part by the maternal immune system after conception [56]. In humans, one groups has evaluated the methylome of over 300 pregnant women using a high throughput DNA methylation analysis and found that a score of differentially methylated regions better predicted atopic disease in children than clinical data [57]. The same group is currently looking at the correlation of DNA methylation in the neonates cord blood to better understand the mechanisms involved.

### Transgenerational inheritance

It has been well described that through development, with the shift from pluripotent stem cells to well differentiated specialized cell types, chromatin becomes increasingly repressed by histone modifications and less activated by permissive histones [58]. However, while it was originally thought that epigenetic marks were completely erased from germline upon conception, this concept has been disproved over a decade ago [59,60]. It is now evident that epigenetic changes induced by environmental exposure may alter the epigenome of the germline and persist through generations.

In their canonical experiment with the agouti mouse model, Morgan et al., fed mice with a methyl-donor rich diet that favored the methylation of the agouti gene, which codes for signalling peptide in mice, which affects coat colour pigmentation. Not only did this influence their immediate offspring’s fur color, but throughout five generations with the exact same diet, the fur coat would get darker and darker showing transgenerational transmission of the phenotype, which is not only inherited but augmented [60]. The experiment was repeated recently by Cropley and colleagues who studied the effect of stopping the methyl donor rich diet after 5 generations [61]. Interestingly they found that the following generation (F_6_), which was not exposed to the diet, actually exhibited a further increase in fur pigmentation, with normal color returning only with the second generation off therapy (F_7_). Mice from the fifth generation had been weaned and put on normal control diet before mating, showing the effect of the diet did not take place in utero. Rather, it suggests that germ cells exposed to excess methyl donors within the developing F_5_ females retained a memory of the methyl donor effect, manifested in F_6_ mice [61].

In utero diet supplementation with methyl donors has similarly been shown to increase allergic disease in a mouse model [62]. The F1 progeny exposed to an in utero diet supplemented with methyl donors demonstrated enhanced cardinal features of allergic airway disease, including airway hyperreactivity, lung lavage eosinophilia and IL-13, higher concentrations of serum IgE as well as change in splenocyte phenotype compared to controls on normal diet. More importantly, these traits were passed down transgenerationally, although somewhat less robustly, in F2 “grand-children” mice which did not have in utero supplementation. In human, transgenerational inheritance is exemplified by the effects of tobacco, which may last for 2 generations. Li et al. compared 338 children diagnosed with asthma in their first 5 years of life to 570 countermatched controls and found that a child whose maternal grandmother smoked during pregnancy had double the chance of developing asthma [63]. This risk was even greater if the mother also smoked during pregnancy (OR = 2.6, compared to 1.8 if she did not) supporting the epigenetic transgenerational model in which persistent exposure leads to inheritance and augmentation of the phenotype. However, this association was only replicated for paternal grandmother smoking (which had not been investigated in the former report) in a recent study [64]. The reason for these discordant results is unclear. It could relate to differences in study populations. The first study included children from a southern California cohort [65] (66% white, relatively high levels of air pollution) with slightly earlier diagnosis of asthma (before 5 years) while the second one, from Avon, UK (96% white), included subjects with diagnosis before 7 years of age.

**Figure 5:**
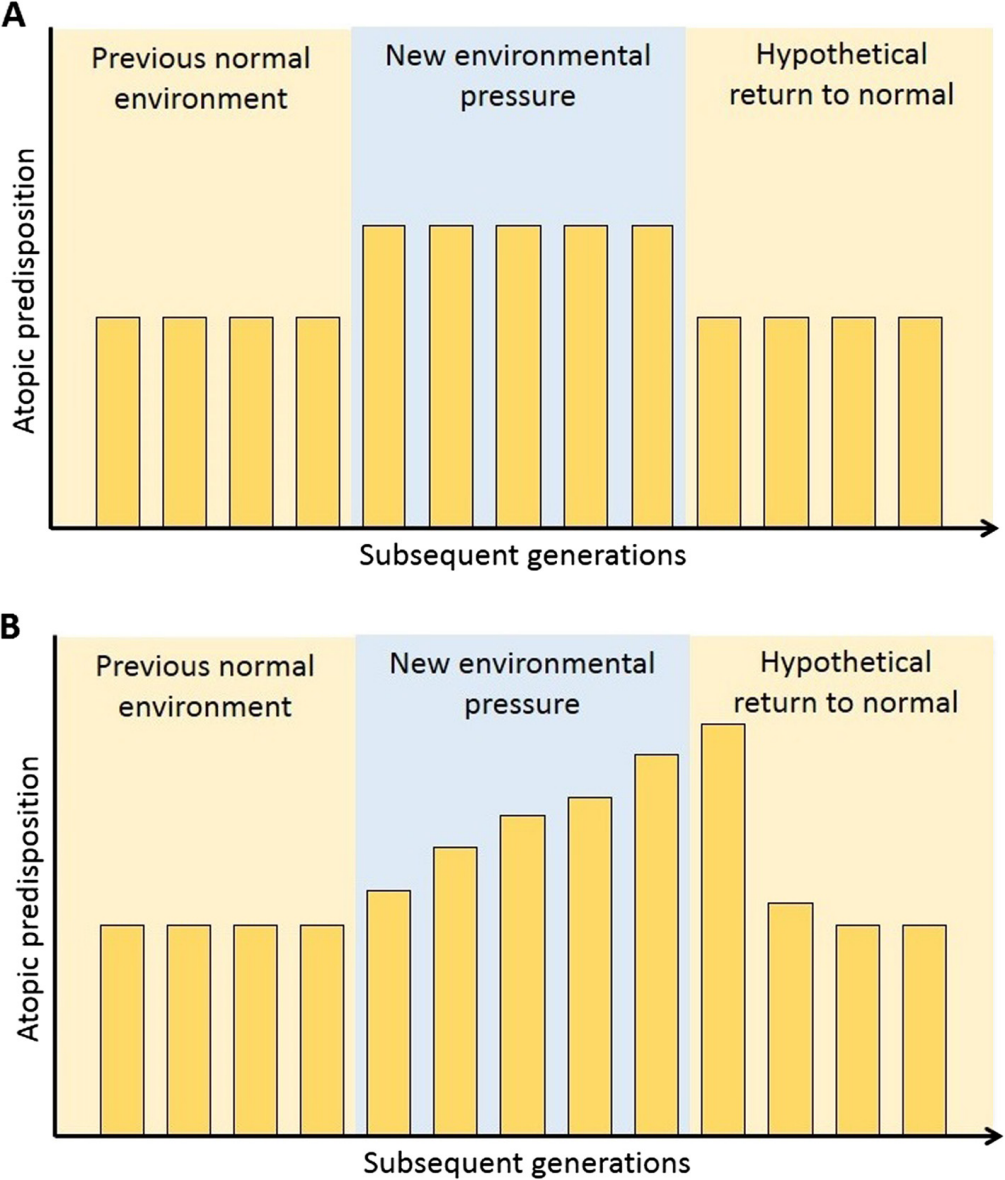
**Transgenerational amplification hypothesis.** Panel **A** depicts a purely environmental model for the rise of atopic disease, in which a change in environment increases baseline genetic risk for the disease. Panel **B** depicts an epigenetic transgenerational inheritance model, in which a persistent change in environment does not only increase baseline risk but induces transmittable epigenetic changes leading to amplification of the phenotype with every subsequent generation. Return to a normal environment, will lead to resolution of the phenotype but only after 2 generations.

Transgenerational inheritance of epigenetic traits is extremely interesting from an epidemiologic point of view as it provides a new hypothesis for the persistently increasing prevalence of allergy and related disorders. If the recent increase had been due to a change in environment, prevalence should have increased at once and remained stable as long as this environment remained the same. However, if the new environment induces epigenetic changes, a transgenerational amplification of the atopic phenotype would be expected even with stable exposure (Figure 5). Furthermore, according to this hypothesis, it would be expected that the benefit of some interventions to prevent allergies (such as pro- and prebiotics) could take a full generation before reaching their full effect, hence possibly the somewhat disappointing results so far [66].

## Association studies in asthma and allergy

At this stage, most of the epigenetic literature on asthma and allergy consists of association studies [67-88]. As with genetic association studies, both candidate gene and genome-wide approaches are used. Candidate gene approach has the benefit of allowing the specific study of a certain number of particularly relevant genes. Its main disadvantage is the fact that it relies on the investigators hypothesis as to which gene(s) to study. Genome-wide approaches do not have this limitation. However, the amount of analysed data is so great that some relevant but weakly associated loci may be lost after statistical corrections.

**Figure 6:**
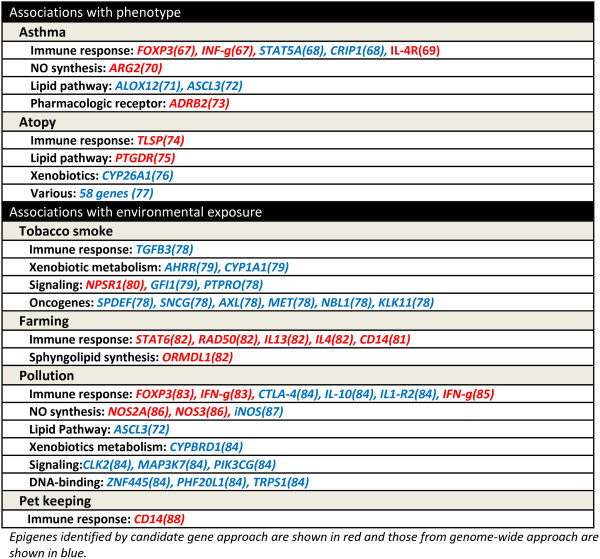
Loci identified from previous DNA methylation association studies for asthma, atopy and related exposures.

Figure 6 provides a list of selected loci that have been reported to be associated with allergic phenotypes and/or environmental risk factors. Association studies are extremely helpful to provide a description of the epigenetic landscape of a given phenotype, which can lead to the identification of potential biomarkers or targets for therapy. However, they are not sufficient to conclude causality, especially when dealing with a mixed cell population (biopsy or whole PBMCs). Since different differentiated cell types exhibit a different methylation pattern (i.e. Th2 vs Th1 vs Treg cells), changes in the proportion of these subsets will directly affect the methylation pattern of the overall population. Thus, finding more demethylation of the Th2 LCR genes (IL-4, IL-13) in PBMCs from atopic individuals may be only a reflection of the larger proportion of Th2 cells in those individuals, and not the cause of it.

An exciting aspect of epigenetics is that it sheds a new light on previous genetic association studies. Since epigenetic marks have the potential to silence a gene, it is to be expected that it can modulate the effect of an underlying polymorphism on disease risk. For example, IL-4R single-nucleotide polymorphism (SNP) rs3024685, which is not associated with asthma on its own, carries a significant risk for the disease when controlled for IL4-R methylation [69].

Similarly, the 17q12-21 which carries 2 polymorphisms associated with maternally inherited risk for asthma was found to be highly methylated in adult males [89]. In fact, epigenetic changes can alter the effect of gene polymorphisms over time. The decreasing effect of CD14 polymorphism on soluble CD14 levels has been shown to be paralleled by small but significant increases in CD14 methylation from 2 to 10 years of age [90].

Genetic polymorphisms can also in turn affect epigenetics regulation of gene expression at a given locus [71,75]. Morales and colleagues showed that hypomethylation of CpG site in the arachidonate 12-lipoxygenase (ALOX12) gene correlated with wheezing in two Spanish cohorts. In both cohorts, they found that the extent of hypomethylation correlated with the genotype for haplotype-tagging single nucleotide polymorphism (SNP) rs312466. In the Menorca cohort for example, subjects with G/G genotype had a 25% genome methylation at CpG site E85, compared to 15% and 7% methylation for genotypes G/A and A/A, respectively) [71]. Recently, North and colleagues found that DNA methylation of the Ephrin-B3 gene (EFNB3), a transmembrane ligand for receptor tyrosine kinases involved in bidirectional signaling, correlated with total symptom scores recorded after exposure to grass for two days in an Environmental Exposure Unit [91]. They also identified two SNPs which influenced on methylation status. One of those, rs3744262 changes a cytosine for a thymine on a CpG site (CpG-SNP) and therefore directly impacted on its methylation. These studies show how polymorphisms and epigenetic regulations are interrelated and how future studies should be structured to examine these interactions.

### Potential underlying mechanisms

Although our understanding is still superficial, there is an increasing amount of literature looking into the mechanisms by which allergic phenotypes and environmental exposures could be associated with specific epigenetic changes.

In lung biopsies, the HDAC: HAT ratio has been shown to be lower in asthmatic samples and to correct with treatment [92-96]. This is significant as endogenous HDAC activity may play a crucial role in maintaining the balance of pre-established Th1-like and Th2-like responses. When their endogenous HDAC activity is inhibited, ex vivo memory T cells show an increase in Th2-associated recall response (IL-13, IL-5) and reduction in Th1- (IFN-γ, CXCL10) or Tr1-associated (IL-10) recall response, shifting the Th1:Th2 ratios by 3-fold to 8-fold [97]. However, the relation between HDAC/HAT activity and allergic inflammation is not that simple. Inhibition HDAC may also increase the suppressive function of FOXP3+ Tregs ex vivo [98] and the induction of pTregs by the metabolites of commensal bacterias has been shown to relate to their HDAC inhibitory property which decreases proinflammatory cytokine expression in dendritic cell [99]. The transmaternal asthma protection provided by the farm-derived gram-negative bacterium Acinetobacter lwoffii F78 was associated with an increase in permissive H4 acetylation of the INF-γ locus and this protection was abolished when mice were treated with an HAT inhibitor [100]. It is important to point out that histones are not the only targets of acetylation. Multiple cellular proteins with important functions are also regulated by acetylation and will be affected by changes of HDAC/HAT activities. This includes signal transducers and nuclear factors that are relevant to the immune response such as GATA-3 and FOXP3 [101]. More specifically, mice studies have shown that optimal Treg function requires acetylation of several lysines in the forkhead domain of Foxp3 which enhance binding to the Il2 promoter and suppress endogenous IL-2 production [102,103]. In the end, the question remains as to what causes this change in HDAC/HAT activity in the first place in atopic subjects.

Environmental tobacco smoke supresses the expression and activity of HDAC2 and HDAC3 [104,105]. Exposure to tobacco smoke has also been shown to alter the expression of dnmt1 and dnmt3b [106]. Both tobacco smoke and pollution induce oxidative stress which are thought to favour the demethylation process, as well as to cause lesions to DNA which prevent binding of Dnmt, resulting in a non-specific decrease in methylation across the genome [107-109]. This non-specific interference with the epigenetic process could potentially have a major impact on a concomitant immune response to an allergen given the complex epigenetic control involved in mounting such a response.

Although a diet rich in methyl-donor nutrients has been shown to promote DNA methylation and to induce an allergic phenotype in mice, the same has yet to be shown for humans [62]. Maternal intake of folic acid (a methyl donor) during pregnancy does not influence risk for atopy [110], asthma [111] or food allergy [112]. Whether the previously reported protective effect of antioxidant supplement can be related to an effect on DNA demethylation is still undetermined [113-116].

## Conclusion

Epigenetics is an exciting new field in allergy and asthma research that has strongly evolved in the last decade. Recent studies shed a new light on the pathogenesis of this complex group of disease, not only with regards to gene-environment interaction but also with regards to the model of inheritance and its epidemiological implications. The field is still at its infancy stage and more work needs to be done to dissect the epigenome of asthma and allergy and to better understand its underlying mechanisms.

## Abbreviations

DNMT: DNA methyltransferase; HAT: Histone acetyltransferase; HDAC: Histone deacetylase; TET: Ten-eleven translocation; DHS: DNAse I Hypersensitivity site; TSDR: Treg-specific demethylated region; LCR: Locus control Region; TCR: T cell receptor; SNP: Single nucleotide polymorphism.

## Competing interests

The authors declare that they have no competing interests.

## Authors’ contributions

PB performed litterature review and wrote the manuscript. NK reviewed the manuscript. Both authors read and approved the manuscript.
